# Nucleus Basalis of Meynert Stimulation for Dementia: Theoretical and Technical Considerations

**DOI:** 10.3389/fnins.2018.00614

**Published:** 2018-09-03

**Authors:** Deepak Kumbhare, Viktoras Palys, Jamie Toms, Chathurika S. Wickramasinghe, Kasun Amarasinghe, Milos Manic, Evan Hughes, Kathryn L. Holloway

**Affiliations:** ^1^Department of Neurosurgery, Virginia Commonwealth University Health System, Richmond, VA, United States; ^2^McGuire Research Institute, Hunter Holmes McGuire VA Medical Center, Richmond, VA, United States; ^3^Department of Neurosurgery, University of Arkansas for Medical Sciences, Little Rock, AR, United States; ^4^Southeast PD Research, Education and Clinical Center, Hunter Holmes McGuire VA Medical Center, Richmond, VA, United States; ^5^Department of Computer Science, Virginia Commonwealth University, Richmond, VA, United States; ^6^School of Medicine, Virginia Commonwealth University, Richmond, VA, United States

**Keywords:** basal nucleus of Meynert, Parkinson’s disease dementia, deep brain stimulation, diffusion tensor imaging, neuronal oscillations, quantitative electroencephalography

## Abstract

Deep brain stimulation (DBS) of nucleus basalis of Meynert (NBM) is currently being evaluated as a potential therapy to improve memory and overall cognitive function in dementia. Although, the animal literature has demonstrated robust improvement in cognitive functions, phase 1 trial results in humans have not been as clear-cut. We hypothesize that this may reflect differences in electrode location within the NBM, type and timing of stimulation, and the lack of a biomarker for determining the stimulation’s effectiveness in real time. In this article, we propose a methodology to address these issues in an effort to effectively interface with this powerful cognitive nucleus for the treatment of dementia. Specifically, we propose the use of diffusion tensor imaging to identify the nucleus and its tracts, quantitative electroencephalography (QEEG) to identify the physiologic response to stimulation during programming, and investigation of stimulation parameters that incorporate the phase locking and cross frequency coupling of gamma and slower oscillations characteristic of the NBM’s innate physiology. We propose that modulating the baseline gamma burst stimulation frequency, specifically with a slower rhythm such as theta or delta will pose more effective coupling between NBM and different cortical regions involved in many learning processes.

## Introduction

Despite promising results from a single patient with PDD and stimulation of NBM, two small trials of NBM stimulation in AD and PDD have provided limited benefits (Freund et al., 2009; Kuhn et al., 2015a; Gratwicke et al., 2018).

The original case report described the combined stimulation of the STN and NBM nuclei in a 71-year-old man with slowly progressive Parkinson-dementia syndrome. Turning on electrical stimulation of the NBM at 20 Hz resulted in improvement in attention, concentration, alertness, drive, and spontaneity. This resulted in the patient’s renewed enjoyment of former interests and enhanced social communication. In a follow-up report, the authors describe the restoration of the patient’s ability to perform ADLs and activities of interest due to improvement in apraxia. The apraxia only improved with activation of the NBM leads but not the STN leads. This improvement was seen in the patient’s normal daily activities as well as on the FAST.

Subsequently, six patients (4F/2M), with AD (Mental State Examination, MSE scores 18–26), aged 57–79 were enrolled in a phase II study to evaluate the safety and feasibility of bilateral low-frequency DBS of the NBM for treatment of their dementia. Although the goal was to implant in the ch4-im and ch4-p segments of the NBM, atrophy, lacunes and vessels precluded this and so the leads were placed in a more broad area as dictated by patient anatomy. Stimulation consisted of activation of the distal contacts at 2–4.5 v, 90–150 PW, and 20 Hz. During a 4-week double-blind sham-controlled phase and a subsequent 11-month follow-up open label period, clinical outcome was assessed by using the ADAS-cog as the primary outcome measure. Electroencephalography and [(18)F]-fluoro-deoxy -glucose PET were secondary endpoints.

The ADAS-cog score improved in 1 patient and was stable in 3 patients at the 1-year endpoint. In this progressive disease, the group mean demonstrated a worsening of 3 points over the course of the year, which is better than the worsening score of 4.5 seen in a large long-term study of progression of AD in patients treated with anti-dementia drugs (Scarmeas et al., 2007) and the 4.2 point worsening seen in the fornix DBS trial (Kurz et al., 1999). Although four of the six patients were considered responders, only one patient improved and 3 had slightly better outcomes than participants on anti-dementia drugs. Two of the patients did not respond.

The same authors tested the hypothesis that younger and less affected patients would benefit more from NBM DBS for the cognitive decline associated with AD. They implanted 2 additional patients who were younger, aged 61 and 67 years old, with better (lower) ADAS-cog scores of 9 and 11 in the NBM, and applied stimulation of 20 Hz without a cross over design. The patients’ baseline scores were compared with those at 1 and ∼2 years after implantation. One had stable to improved scores at 1 and 2 years. The second patient declined in cognitive scores but normalized his clock agnosia score.

Six years later, the 2 youngest patients in the aforementioned studies participated in an EEG evoked response study. The other 6 patients had progressed too much to participate.

In their most recent publication, the authors reviewed the postoperative outcome at 6 and 12 months in 10 patients with NBM DBS for AD (including the 8 discussed above). The MMSE stabilized and a non-significant slight improvement was seen in the first 12 months. A similar trend can be observed for the ADAS-mem, with a non-significant progressive improvement of the values. In the ADAS-cog, a continuous non-significant decline in cognitive function was observed. The locations of the DBS contacts were analyzed with respect to each of these outcome measures. Each outcome measure was correlated with a different location. Better outcomes on the MMSE were associated with more ventral/caudal locations, while better ADAS-mem outcomes were associated with posterior locations, and more anterior locations favored better ADAS-cog outcomes. This suggests the presence of multiple circuits differentially affected by lead location. Preoperative preservation of cortical thickness in the frontal-parietal-temporal cortex was associated with better outcomes.

In Gratwicke et al. (2018), six patients who were appropriate candidates for DBS surgery to treat their motor symptoms of PD, except that they also suffered from PDD, were offered participation in a RCT blinded trial of NBM stimulation with the intent to treat the motor symptoms with alternate contacts after trial completion. The patients were implanted with bilateral DBS leads spanning the motor GPi and the underlying NBM in the region of ch4i. The entry site was altered so that the trajectory was nearly perpendicular to the ac-pc plane. Ch4i was chosen both for its proximity to the motor GPi as well as its widespread cortical projections. The leads were placed with a Leksell frame under general anesthesia without MER and the location was immediately confirmed with a postoperative MRI. The patients tolerated the procedure well, with return to baseline orientation within 48 h. This was despite having a range of DRS scores from 101 to 126, mean = 114.2 (SD 9.7).

The patients’ leads were mapped at 1 month after surgery. The mapping was conducted with 20 Hz, 60 PW, and monopolar stimulation and consisted of choosing the contact and voltage that produced the highest digit spans at the lowest voltage without adverse effects. The device was then not activated for another 2 weeks. At 6 weeks half the patients were randomized to active stimulation for 6 weeks and the others to sham stimulation. At the end of this, there was a 2-week washout period followed by crossover to the other group. Multiple neuropsychiatric assessments were utilized including the DRS-2, FAST, California Verbal Learning Test, and the NPI. Only dyskinesias and the NPI improved with stimulation on. The NPI improvement was due to a marked improvement in hallucinations and resultant caregiver distress in 2 of the patients. Interestingly, despite the use of the digit span to map the contacts, this did not improve in the population and was worse on NBM stimulation than off, although this did not reach significance (*p* = 0.07).

In summary, NBM stimulation for the treatment of dementia in AD and PDD was well tolerated and had dramatic effects in a few of the individuals treated, but not the majority. Within the PDD group, the index patient and the 2 patients in the Gratwicke trial that had improvement were due to changes in parietal symptomatology. Constructional apraxia and visual hallucinations are both tied to parietal lobe function. There are several factors that have not been fully explored in these prior studies that have a likelihood of significant impact on the outcome. In this article we explore stimulation parameters, targeting, and programming guidance as potential means of optimizing the efficacy of NBM stimulation for better outcomes.

The promise of NBM stimulation can be inferred from a long history of investigation as a research tool to study the mechanisms of learning and memory. While the NBM innervates most of the cortex, the hippocampus, however, receives its ACh input from the medial septum rather than the NBM. Thus, most studies of NBM have focused on neocortical plasticity in such regions as auditory, somatosensory, and visual cortex. The inherent activity of the NBM as well as NBM stimulation results in the release of ACh, altered neocortical excitability, modulation of the level of synchrony in neocortex, and enhanced synaptic plasticity. These processes are known to be intrinsic to the formation of cortical memory engrams. During reinforced learning, NBM activity induces synaptic plasticity and enhanced representation of the selected stimulus within the relevant cortex as evidenced by increased gamma oscillations. NBM stimulation can, in fact, substitute for reinforcement of the stimulus in the learning process (McLin et al., 2002a; Weinberger et al., 2009, 2013; Miasnikov et al., 2011). The effect of manipulation in NBM has been broadly demonstrated in auditory, somatosensory, visual, and spatial memory sub-circuits (Bakin and Weinberger, 1993; Bjordahl et al., 1998; Kilgard and Merzenich, 1998; Dimyan and Weinberger, 1999; Miasnikov et al., 2001).

Multiple investigators have lent evidence to the hypothesis that activity of the NBM is triggered by stimuli deemed important enough to result in cortical reorganization and that NBM activity is integral to the process of cortical reorganization, whereas the sensory stimulus and the cortex involved determine the engram created (Richardson and DeLong, 1991; Pirch, 1993; Whalen et al., 1994; Maho et al., 1995). The level of detail of the engram is increased by increasing NBM activity (Weinberger et al., 2006). This results in an increase in gamma power in the involved cortex. In the tonotopic auditory cortex, these gamma oscillations are geographically specific enough to mark the tone that has been remembered. Importantly, NBM stimulation has been shown to have a greater effect on memory consolidation in animals with an initial lower learning ability (Butt et al., 2003), which would suggest a more powerful effect in demented subjects.

**FIGURE 1:**
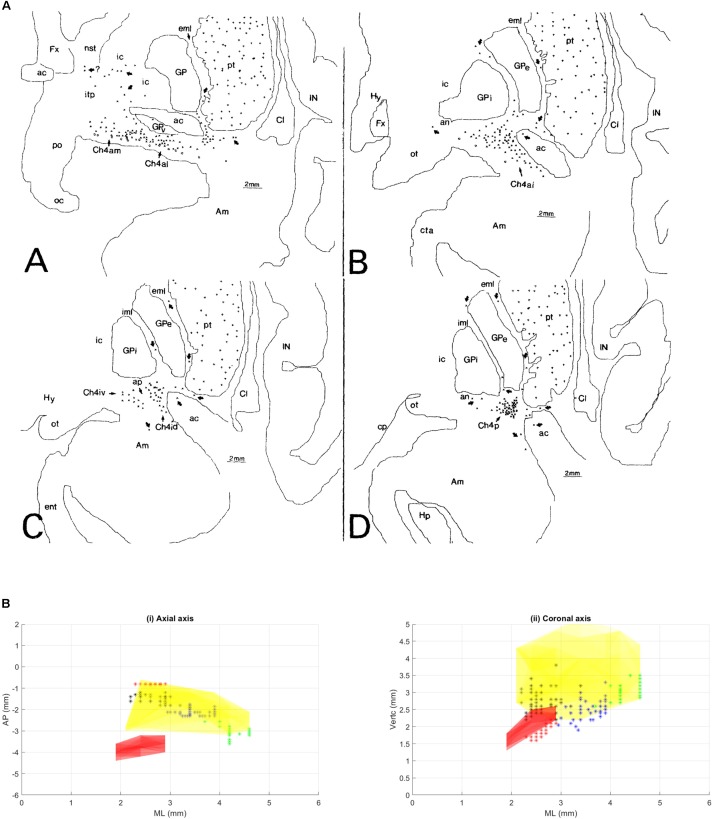
**(A)** This figure was originally published in Mesulam and Geula (1988). These coronal slices of the human brain from Mesulam and Geula (1988) demonstrate the locations of the ChAT positive neurons in the sub-nuclei of the Ch4 complex (NBM) within the context of the basal ganglia anatomy. Permission was granted by John Wiley and Sons (license number: 4330331169891). ac, anterior commissure; Am, amygdaloid nuclei; an, ansa lenticularis; aP, ansa peduncularis; Ch4a, anterior sector of Ch4; Ch4ai, anterointermediate sector of Ch4; Ch4al, anterolateral subsector of Ch4; Ch4am, anteromedial subsector of Ch4; Ch4id, interomediodorsal subsector of Ch4; Ch4iv, interomedioventral subsector of Ch4; Cl, claustrum; cp, cerebral peduncle; eml, external medullary lamina of the globus pallidus; ent, entorhinal cortex; Fx, fornix; GP, globus pallidus; GPe, globus pallidus (external segment); GPi, globus pallidus (internal segment); Hp, hippocampal formation; Hy, hypothalamus; IC, internal capsule; iml, internal medullary lamina of the globus pallidus; IN, insular cortex; itp, inferior thalamic peduncle; nst, nucleus of the stria terminalis; oc, optic chiasm; ot, optic tract; PO, preoptic area; pt, putamen. **(B)** Illustration of different NBM territories in the axial **(i)** and coronal **(ii)** plane, based on the range of coordinates described in the following NBM studies in rats. Boix-Trelis et al. (2006) observed increased C-Fos expression in cingulate, parietal, piriform and perirhinal cortices, but not in entorhinal cortex or amygdala nuclei when stimulated NBM sub regions around 0.95–1.8 mm posterior to bregma and 2.8 mm lateral to midline (black asterisks) (Boix-Trelis et al., 2006). Weinberger et al on the other hand showed that the activity of NBM in posterior 2.3 mm and lateral 3.3 mm (blue asterisks) is strongly correlated to auditory cortex (Weinberger et al., 2009, 2013). On the other hand, the barrel cortex is more predominantly affected by NBM stimulation in the region posterior 0.8 mm, L 2.6 mm (red asterisks). Visual cortex, has been estimated to be influenced by an NBM sub-region around posterior 2.8 and lateral 4 mm (Goard and Dan, 2009) (green asterisks). Buzsáki and Wang (2012) have noted that the NBM stimulation changes in EEG are in agreement with the anatomical findings, which provides additional evidence that the NBM projection to the cortex is topographically specific. The GP and STN region (yellow and pink regions respectively) are also shown for reference.

**FIGURE 2:**
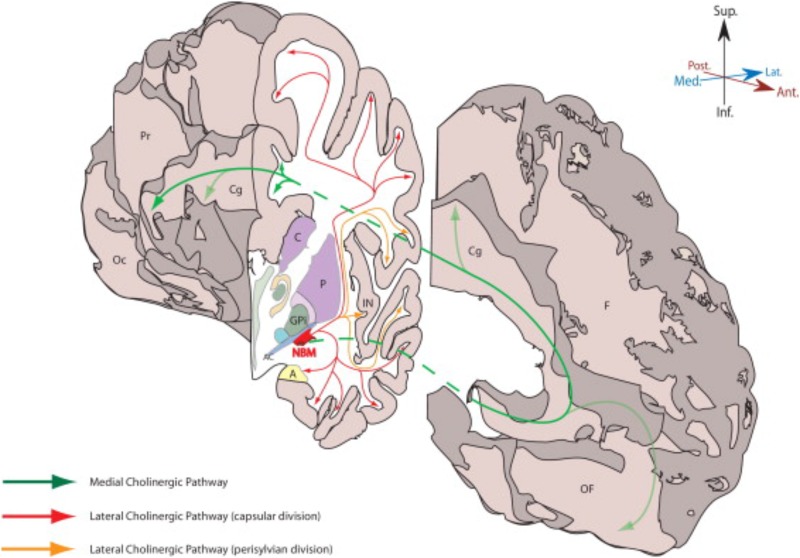
This figure from Gratwicke et al. (2013) demonstrates the NBM and its major cholinergic pathways in human brain. A, amygdala; AC, anterior commissure (lateral aspect); C, caudate; Cg, Cingulate gyrus; F, frontal lobe (medial surface); GPi, globus pallidus (internus); IN, insular cortex; NBM, nucleus basalis of Meynert; Oc, occipital lobe (medial surface); OF, orbitofrontal cortex; P, putamen; Pr, parietal lobe (medial surface). Permission was granted by Elsevier (license number: 4325321354014).

Taken as a whole, the NBM has a clear role in multiple aspects of cognition including arousal, attention, memory, praxis, perception, drive, and spontaneity. Because of the NBM’s widespread connections, there can be significant variability in the effect of its activity or response to stimulation. Thus, the NBM has been divided into subnuclei whose efferent connections have been identified (**Figures 1, 2** and **Table 1**). Stimulation of the distinct NBM projections to medial temporal structures, the amygdala, frontoparietal cortex, or temporo-parietal association areas will enhance cholinergic activity in these cortical regions and thereby will improve mnemonic, attentional or perceptive abilities respectively (Gratwicke et al., 2013). The sublenticular basal forebrain structures, and NBM subnuclei in particular, lack a clear anatomy on conventional neuroimaging due to the limited contrast and spatial resolution. Additionally, analysis of cytoarchitectonic maps demonstrate high inter-subject variability of the sub-compartments of the sublenticular basal forebrain and their stereotaxic position relative to other brain structures (Zaborszky et al., 2008). We propose that DTI can be utilized to better identify the nucleus and target specific cortical regions in order to optimize, or at least, better understand outcomes.

**Table 1:** Nucleus basalis of Meynert (NBM) projections.

NBM and surrounding BF sub-nucleus	Regions	Major projection directed to	Lesser projections directed to
Ch4am	Nucleus basalis of Meynert (NBM)	To medial wall of the hemisphere of frontal, parietal, and cingulate cortices	hypothalamus hippocampal formation ventral somatosensory cortex amygdala ventrolateral orbital middle insular periarcuate peristriate region of occipital cortex, parahippocampal regions inferior parietal lobule
Ch4al	NBM	frontoparietal opercular regions & amygdala.	olfactory bulb medial frontal pole dorsomedial motor cortex ventrolateral orbital cortex insula inferotemporal area parahippocampal regions
Ch4id and Ch4iv	NBM	ventrolateral orbital insula periarcuate peristriate inferotemporal parahippocampal inferior parietal lobule	medial frontal pole dorsomedial motor cortex frontoparietal opercular areas amygdala anterior auditory cortex temporal pole
Ch4p	NBM	Superior temporal gyrus and the temporal pole	adjacent inferotemporal and posterior insular regions
Ch1	Medial septum	Hippocampal complex	orexin/hypocretin neurons in the lateral hypothalamus
Ch2	Vertical limb of the diagonal band nucleus	Hippocampal complex	orexin/hypocretin neurons in the lateral hypothalamus
Ch3	Horizontal limb of the diagonal band nucleus	Olfactory bulb piriform and entorhinal cortices	Orexin/hypocretin neurons in the lateral hypothalamus

***Sources:** (Jones et al., 1976; Mesulam et al., 1984; Mesulam and Geula, 1988; Selden et al., 1998; Simić et al., 1999; Sakurai et al., 2005; Gratwicke et al., 2013; Lun Liu et al., 2015).*

Both in AD and PDD, there is gradual loss of the cholinergic tracts of the NBM. In PDD this is primarily due to neuronal cell loss, whereas in AD this is due to axonal die back (Candy et al., 1983; Perry et al., 1985). This results in pruning of the cholinergic input to the cortex disrupting the spatiotemporal flow of ‘normal’ signals. The flow of information is primarily based on the anatomical connectivity, synaptic strength of the connections, and the selective intrinsic excitability of the network neurons. Thus, optimal stimulation parameters should be designed to rebuild and reinforce this neocortical neuroplastic network. The stimulation patterns can be further modulated to enhance the selectivity of information flow and to establish functional coupling between anatomically segregated regions. Animal studies have demonstrated that NBM stimulation boosts the release of Ach, NGF, and intracellular calcium concentration in astrocytes in the neocortex and amygdala, triggering neuro-plastic changes in the cortex. Thus, this provides a unique opportunity to assess the ability of electrical stimulation to protect or repair ongoing damage to the brain. This is particularly relevant as DBS of NBM aims to stimulate a degenerating nucleus, which is in contrast to traditional DBS targets that involve the targeting of downstream affected circuits. Thus, the selection of stimulation parameters should be evaluated for their potential to regenerate damaged networks as well as for the immediate behavioral response. This regenerative potential may be unique to the NBM and a critical aspect for the success of this approach. Importantly, longer timelines will be required to see a clinical effect of this regenerative potential, if present.

The clinical pilot studies were conducted with continuous tonic stimulation and yet, the vast majority of studies demonstrating learning in animals were conducted with short burst stimulation (**Table 2**). The value of burst stimulation is coming into focus, as recent evidence has demonstrated the critical role of network oscillations in brain function and their origin in neuronal burst firing (Buzsáki and Wang, 2012; Akam and Kullmann, 2014; Fountas and Shanahan, 2017; Hauk et al., 2017; Headley and Paré, 2017; Spitzer and Haegens, 2017). During the learning process, population coded neuronal oscillations vary systematically across space and time. Thus, the integration of the DBS signal into the relevant oscillatory pattern must be considered. We propose that a return to the burst parameters utilized in the animal work has a strong theoretical underpinning, as well as proven efficacy. We also explore another neglected aspect of stimulation parameters: the role of continuous versus task specific stimulation and the role of sleep related NBM activity in the learning process.

**Table 2:** NBM stimulation modes and patterns tested in various learning paradigms in the animal literature.

Article/Research team	Species	Coordinates^∗^	Learning area/s	Stimulation parameters			
				Timing	Amplitude	Mode	Parametersˆ
Kurosawa et al., 1989	Fischer rats	AP: −2.3 mm ML: 3.7 mm DV: −7.6 mm	Ach release in cortex	10 min	200 – 500 μA	Tonic	Tonic gamma *F* = 50 Hz (1–100 Hz) PD = 0.5 ms
Kimura et al., 1990	Wistar rats	AP: −2.3 mm ML: 3.7 mm DV: −7.6 mm	Glucose utilization in cortex	45 min	200 μA	Tonic	Tonic gamma PD = 0.5 ms *F* = 50 Hz (γ)
Adachi et al., 1992	Wistar rats	AP: −2.3 mm ML: 3.7 mm DV: −7.6 mm	Cortical cerebral blood flow	10–60 s	200 μA	Tonic	Tonic gamma PD = 0.5 ms IBF = 50 Hz (γ)
Hotta group: Hotta et al., 2002, 2004, 2007	Wistar rats	AP: −2.3 mm ML: 3.7 mm DV: −7.6 mm	Vasodilation in cortex	11.5–15 min	200 μA	Burst	Delta-gamma CFC ITF = 2 Hz (δ) TD = 1 s PD = 0.5 ms, IBF = 50 Hz (γ)
Hotta et al., 2009	Wistar rats	AP: −2.3 mm ML: +3.7 mm DV: −7.6 mm	NGF secretion induced by NBM stimulation	100 min	200 μA	Burst	Slow and gamma CFC ITF = 0.33 Hz TD = 1 s PD = 0.5 ms IBF = 50 Hz (γ)
Boix-Trelis et al., 2006, 2009	Wistar rats	AP: −1.10mm; ML: 2.8mm, DV: −7.6mm	Socially transmitted food preference and c-Fos expression	Single 20-min session. Or 20 min immediately before the social training	100 μA	Burst	Delta-gamma CFC ITF = 1 Hz (δ) TD = 500 ms IBF = 100 Hz (γ) PD = 0.5 ms
Montero-Pastor group: Montero-Pastor et al., 2001, 2004	Wistar rats	AP: −0.8 to −2.2 (-1.30mm) ML: 2.8mm DV: −7.6mm	Active avoidance acquisition, retention, and retrieval	At different stages of memory formation of the conditioning. For 30–45 min	60–100 μA	Burst	Delta-gamma CFC ITF = 1 Hz (δ) TD = 500 ms IBF = 100 Hz (γ) PD = 0.5 ms
Kilgard group: Kilgard and Merzenich, 1998; Puckett et al., 1998	Sprague Dawley rats	AP: −2.3 mm ML: 3.3 mm DV: −7 mm	Plasticity and re-organization of auditory cortical map.	Paired with auditory tone (5 ms after tone) occurred randomly every 8–40 s. Repeated for 300–500 times per day for 20–25 days	70–150 μA. biphasic pulses	Paired burst	Single gamma burst TD = 250 ms IBF = 100 Hz (γ) PD = 0.1 ms biphasicˆ
Bao et al., 2003	Sprague Dawley rats	AP: −2.3 mm ML: 3.3 mm DV: 7.0 mm	Changes in acoustic representation in auditory cortex.	initiated 200 ms after sound onset,	100–200 μA	Paired burst	Single gamma burst TD = 0.2 s IBF = 100 Hz (γ) PD = 0.1 ms biphasic pulses
Weinberger group: Miasnikov et al., 2006; Miasnikov et al., 2011	Sprague–Dawley rats	AP: 2.3 mm ML: ∼1.46 mm DV: ∼-8 mm	Alterations in auditory EEG spectrum and behavioral frequency generalization gradients	Paired/followed with auditory tone.	100 μA	Paired burst	Single gamma burst TD = 0.2 s IBF = 100 Hz (γ)
Howard and Simons, 1994	Sprague-Dawley rats	AP: −0.8 ML: 2.6 DV: −7 mm	Whisker stimulation and neuronal activity in barrel cortex.	Paired with whisker stimulation (Randomized sequences of the 8 stimulus angles 15 times with a 2 s interstimulus time interval)	1.0–1.5 mA	Paired burst	Single gamma burst TD = 10–20 s PD = 0.5 ms IBF = 50 Hz (γ) train of 50 Hz monophasic square waves.
Berg et al., 2005	Long Evans rats	AP: −2.1 mm ML: 1.2 mm DV: 6.9 mm	Facilitation of vibrissa motor (M1) cortex	Paired with intracortical microstimulation.	100 μA	Tonic	Ultra-high-gamma PD = 200 μs 500 Hz
Goard and Dan, 2009	Long-Evans rats	Based on changes in power spectrum of V1 LFPs.	Stimulation induced inter neuronal decorrelation and enhanced EP in visual cortex	Starts 0.5–1 s before the visual task and is maintained during the task.	Not mentioned	Burst	Gamma burst TD = 500 ms IBF = 100 Hz (γ) PD = 0.1 ms
Lee et al., 2016	Sprague–Dawley rats	AP: −1.32 mm, ML: + 2.8 mm, DV: −7.4 mm	Visuospatial memory tasks (consolidation and retrieval)	Daily for 1 week until the start of behavioral testing (1 h per day, 1 week in total).	1 V (constant voltage)	Tonic	Tonic gamma F = 120 Hz (γ) PD = 90 μs bipolar

*^∗^AP, Anterior-Posterior planes (negative = Posterior to bregma). ML, mediolateral planes (distance from mid line). DV, dorso-ventral planes (negative: depth from dura). ˆITF, Inter-Train Frequency; TD, train duration; PD, pulse duration; IBF, Intra-burst (or train) Frequency. NBM, Basal nucleus of Meynert.*

An additional challenge in stimulation of the NBM is the absence of an immediate behavioral response to guide DBS programming. In the case of motor disorders therapy, the parameters for the stimulation of the sub-regions in STN and GPi are based on the immediate, visible, and measurable effects on PD symptoms such as tremor, rigidity, and bradykinesia. A similar approach is not applicable for NBM DBS treatment of dementia, as the behavioral effects are more subtle and require time to manifest. Both PET and electroencephalography (EEG) have been utilized as secondary outcome measures in the stimulation of cognitive and limbic targets, however, the EEG has the advantage of allowing repeated assessments without radiation exposure and the ability to obtain a real time response to changes in stimulation parameters. Additionally, the EEG response has been correlated with PET metabolic response to stimulation. QEEG involves the mathematical processing of EEG data to allow derivative factors to be extracted from the EEG data. This provides objective data, in contrast to the visual qualitative interpretation of the standard EEG, further enhancing its utility (Cozac et al., 2016).

In summary, we propose that NBM stimulation can be optimized through: (1) targeting through DTI tractography, (2) selection of stimulation parameters that optimize the regenerative/neuroplastic potential of the NBM as well as the oscillatory patterns critical to learning and attention, (3) the use of QEEG as an objective physiologic measure to visualize the effects of stimulation parameter selection in real time.

In this article, we discuss various technical and theoretical considerations to systematically address each of the above factors required to design an optimal NBM-DBS strategy.

## Targeting

Targeting of the NBM is challenging from a surgeons perspective. It is a thin flat nucleus perpendicular to the standard DBS trajectory originating at a frontal burr hole. The NBM has no clear anatomic boundaries and its position can only be inferred by the surrounding structures. Additionally, the nucleus has connections with almost every portion of the cortex but the origins of these connections are invisible on an anatomic MRI. In each of the published DBS of NBM case series, we found that the authors reported stimulating slightly different NBM subregions. Freund et al. (2009) stimulated the dorsolateral portion of Ch4i – the largest subnucleus with the most widespread cortical projections (Mesulam et al., 1984) giving the potential to affect more cognitive domains. The recent study by Gratwicke et al. (2018) targeted a different portion of Ch4i – underneath the traditional GPi target for PD – to allow fallback treatment of motor symptoms. Due to the microcystic degeneration in Alzheimer patients, the Kuhn group (Kuhn et al., 2015a) was forced to target a range of NBM areas, extending from CH4im medially to Ch4p in the subputaminal region and the substriatal terminal island, laterally. Thus, compromises in targeting can explain some of the variability of results in these studies.

The NBM, also known as Ch4, occupies a large portion of the sublenticular basal forebrain and is an “open” nucleus forming several clusters within the basal forebrain (Mesulam and Geula, 1988). In the detailed postmortem human study by Mesulam and Geula (1988), the most anterior islands of NBM appeared in the basal forebrain at a point just caudal to the olfactory tubercle; the nucleus reached its greatest mass under the AC and extended caudally to the level of the uncal hippocampus spanning a distance of 13–14 mm in the anteroposterior axis; in its anterior portion the NBM was limited inferiorly by the horizontal limb of the nucleus of the diagonal band of Broca, superomedially by the ventral globus pallidus, and superolaterally by the lateral extension of the AC. In its posterior portion the NBM abuts the ansa lenticularis superiorly, the putamen laterally, the posterior tip of the amygdala inferiorly, and the optic tract medially (Mesulam et al., 1984; Zaborszky et al., 2008). At its widest mediolateral extent, the nucleus spans a distance of 16-18 mm^2^. The probabilistic stereotactic map of Ch4 by Zaborsky allows the integration of the anatomy within stereotactic space. The data was reported in stereotactic space coordinates based on the midline AC and demonstrated significant overlap across subjects from 0 to 6 mm behind the AC, with the medial to lateral extent occupying the width of the globus pallidus interna/externa (GPi/GPe) complex.

The NBM can be subdivided into regions. The most popular NBM subnuclear nomenclature was introduced by Mesulam et al. (1983). This seminal investigation of the cortical connections of the NBM in the Rhesus monkey provides details on the connectivity of each subnucleus summarized in **Table 1**. The reader is referred to the paper for details, but a simplified summary states that the Ch4am supplies the medial hemisphere, Ch4al supplies the fronto-parietal opercular areas and the amygdala. Ch4i supplies dorsal prefrontal, insular, posterior parietal, inferotemporal and peristriate cortices (lateral parieto-occipital cortex in primates). Ch4p is heavily concentrated in the superior temporal lobe including auditory cortex. In (Mesulam and Geula, 1988), the anatomy of the subnuclei in 16 human cadavers was investigated and is illustrated in **Figure 1A**. An additional sector is delineated in the human. In non-human primates there was a sharp border between Ch4am/al to Ch4iv/id, but in humans this is more gradual. He coined the term Ch4ai to describe this transition area in humans and to maintain consistency of labeling in the other areas amongst all primates.

A similar type of study was conducted by Selden et al. (1998) to identify the major fiber tracts from the NBM and is illustrated in the figure from Gratwicke et al. (2013) and shown in **Figure 2**. This analysis utilized 5 cadaver brains that were stained with cresyl violet for cytoarchitectonic identification and acetylcholinesterase histochemistry and the projections were traced onto an MRI. The cortical projections originate from the distinct subnuclei of NBM and form three major bundles – medial and two lateral (perisylvian and capsular) (Selden et al., 1998). There is a prominent nexus at the lateral aspect of the NBM and another at its anterior extent progressing to the white matter of the gyrus rectus.

The NBM in rodents is even more irregularly dispersed than its human counterpart, but also contains distinct sub-regions. Targeting specific sub-regions of the rodent NBM is based on anatomic coordinates (**Figure 1B**), at times refined with electrophysiology, but routinely confirmed by sacrifice and staining. Obviously, an alternative to sacrifice and staining is required for a clinical trial.

Diffusion tensor imaging may be able to provide *in vivo* tractography maps of NBM subnuclear – cortical connectivity and thereby allow direct visualization of the target of stimulation. DTI has been utilized to study the connectivity and mean diffusivity of the NBM outflow in AD and or PDD, but so far has not been utilized to visualize these tracts in 3 dimensional space. There are significant challenges to this approach. DTI poorly discriminates tracts that include turns of >45° or those that pass-through crossing fibers, features that are found in abundance in the outflow tracts of the NBM. Although this precludes following all of the fiber tracts to their final destination in distant cortical regions, the delineation of the initial segments as they exit the NBM could help to identify the NBM itself as well as sub-regions of cortical connectivity. An additional issue in DTI, is the problem of geometric distortion. Air-tissue interfaces around the sinus air cavities create an inhomogeneous magnetic field corrupting the spatial encoding gradient, resulting in geometric distortion and loss of signal. This distortion can be most easily visualized in the displacement of the DTI generated AC and optic nerves from their anatomic counterparts. This is very relevant to the NBM, which sits directly adjacent to the sinuses and is framed by these 2 structures. Although there are increasing methodologies to correct the distortion, this remains a significant issue in the field. Despite these limitations, DTI can provide information regarding the relative location of the subnuclei within the anatomic structure.

Deep brain stimulation targeting that utilizes DTI information may have several advantages. Various types and subtypes of neurodegenerative processes may affect different NBM subnuclei to a different extent. It may allow targeting of the areas of cortex affected in any given patient as evidenced by their clinical deficits, e.g., parietal cortex for hallucinations and temporal lobe for word finding difficulties. The knowledge of the specific cortical region targeted can also help with the selection of appropriate outcome measures tailored to the corresponding cognitive function inherent to that cortex. Neuronal loss may lead to insufficient critical mass of cholinergic cells as evident by absent projections that can only be revealed by DTI. Lastly, it may be more practical to target the larger projectional fiber tracts than just the small portion of the nucleus accessible with the standard perpendicular approach. DTI can provide *in vivo* tractography maps of NBM subnuclear – cortical connectivity and thereby allow direct visualization of the target of stimulation.

### Preliminary Investigation of DTI Definition of NBM Tracts (Original Data)

We investigated the ability of DTI to identify the efferent/afferent pathways of the NBM with 3 goals in mind: (1) to identify the origins of specific cortical efferents within the nucleus, (2) to evaluate the ability of a DBS electrode originating at a burr-hole created for GPi targeting to intersect with these pathways and their origins within the nucleus in an effort to maximize the utility of the contacts on the lead (3) to evaluate whether the microcystic changes in the NBM of AD patients (described by Kuhn et al., 2015a,b) would also limit NBM targeting in PDD. The overall goal of our study was to identify a location within the NBM that would provide the greatest interaction with the overall nucleus either through cell body or the cortical projections that originate from the distinct subnuclei of the NBM. As previously stated, the limitations of DTI in terms of following tracts through turn of >45 degrees and through crossing fibers preclude identification of the specific cortical target of the fibers.

For our retrospective study (in progress), we randomly selected nine male patients who had DBS surgery at our institution for Parkinson’s disease. All subjects had fluid-attenuated inversion recovery (FLAIR) and diffusion-weighted MR imaging with 8 channel head coils on 3.0 T GE 750 MRI system and neuropsychological evaluation done preoperatively. Diffusion data was collected in 2 mm contiguous axial slices, 60 diffusion-gradient directions, 1 NEX with a *b*-value of 1000 s/mm2 and 5 volumes with no diffusion-weighting and a *b*-value of 0.

Due to the lack of distinct anatomical boundaries in NBM (Mesulam and Geula, 1988), we chose the following anatomical imaging demarcations to define our area of interest, reflecting the approximate boundaries of the NBM area: inferior – the basal surface of substantia innominata containing the anterior perforated space; superior – the bottom margin of lentiform nucleus; medial – the anterior portion of hypothalamus; lateral – the sagittal plane through the lateral edge of lentiform nucleus to include the nucleus subputaminalis (also called Ayala’s nucleus) which represents a subsector of the NBM (Boban et al., 2006); anterior – the coronal plane one full anterior-posterior diameter of AC in front of AC; posterior – the coronal plane through the anterior border of mammillary bodies. Of note, the hand segmentation of the NBM used in our study lacks precision and introduces some variability. This was also noticed in studies of substantia innominata with hand tracings for volume measurements where intra-rater and inter-rater reliability of 0.92 and 0.89, respectively in Choi et al. (2012) and 0.82 and 0.80 in Kim et al. (2011) have been found.

The VTA at 2 volts with standard stimulation parameters is expected to be 2 mm in a radius from the DBS electrode contact center. Therefore, we divided NBM area into 15 equal segments of 4 mm in diameter and recorded their AC-PC coordinates in Talairach and Tournoux stereotactic space. Tractography seedings were done from each segment separately and the analysis of white matter projections was performed utilizing the commercially available deterministic DTI tractography software (FA start value 0.20, ADC stop value 0.10, minimum fiber length 0, and maximum directional change 45°). The tractography was realigned to the anatomic structures using the AC and optic chiasm as anchors.

**FIGURE 3:**
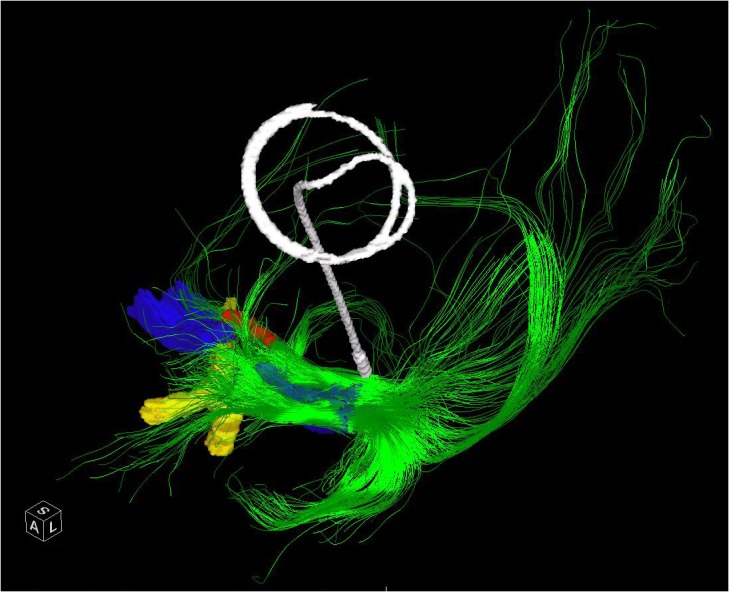
The entire left NBM has been seeded in a patient who subsequently underwent placement of a Left GPI DBS. The post-operative CT has been merged and the lead extracted to demonstrate the relative position of a standard GPI lead in relation to the bulk of the NBM outflow tracts. The bilateral NBM nuclei are reconstructed as described in the text and shown here in blue. The optic chiasm (yellow) and midline aspect of the anterior commissure (red) are shown for perspective. Frontal, temporal (via uncinate fasciculus) and parieto-occipital fibers are visualized.

Many of the projections were truncated at crossing fibers and where > 45 degree turns occurred. Nonetheless the portions that were visible allowed estimation of the lobar distribution of the fibers with caveats, see **Figure 3**. Fibers en passage could not be discriminated and no distinction between amygdala and lateral temporal fibers could be made. Additionally the frontal fibers could not be distinguished from the rest of the medial projection. Lastly, the origin of the medial pathway at the anterior-lateral aspect of the nucleus and the lateral pathways at the lateral aspect of the NBM would be seeded along with the nucleus itself and so the resultant fibers should not be considered specific to this portion of the nucleus. Due to these limitations there is not a one-to-one correlation with the anatomic tracing studies of ACh/NGF fibers described in Mesulam et al. (1983). Nonetheless, the technique does allow for visualization of the origins of these pathways and their ultimate destinations can be interpolated from the anatomic studies. The medial pathway, which travels within the cingulum and supplies the orbitofrontal, subcallosal, cingulate, pericingulate, and retrosplenial cortices was visible as the sole anterior projection. The components of the 2 lateral pathways were difficult to distinguish, except for a tract toward the amygdala and temporal lobe, which followed the uncinate fasciculus trajectory. The parieto-occipital fibers were best visualized due to their relatively straight path. Due to the proximity of the fornix to these regions, sometimes this was included in the reconstructed pathways. This should be considered in targeting of the NBM as it could result in differences in the observed effects of stimulation.

The projections were also quantified by visual rating for fiber density and volume. The analysis was conducted blind to DRS score. The NBM volumes, the heat map of probabilities for the different types of projections, and the resultant patterns were analyzed and correlated with DRS scores and patient’s age. We did not normalize NBM and its fiber volumes to correct for individual brain sizes.

Our DTI analysis, revealed inter-patient and intra-patient (between the right and left sides) variability; nonetheless, several patterns in tractography could be recognized. The anteriorly directed projections that likely contain the components of the medial pathway including the orbitofrontal connections were very specific to the anterolateral sectors of the NBM. In fact, seeds in this area may be picking up these tracts directly. There was a clear projection through the uncinate fasciculus to the temporal lobe, with an origin in the same sector. This likely corresponds to the projection of the NBM to the amygdala and medial temporal lobe. Additional temporal projections could be elicited by seeding the posterior NBM consistent with the Ch4p segment. However, due to truncation of the DTI, we could not ascertain whether the distribution of these fibers was to the supero-lateral aspect of the temporal lobe as would be expected based on the anatomic tracings. The parieto-occiptal projections were more diffusely seeded, consistent with the multiple projections to this broad region, which consists of both the medial and lateral surfaces of the brain. However, the greatest concentration of origins for this region was postero-lateral within the NBM. Although we did not find correlations between the total NBM projectional fiber volume or NBM volume and DRS score in our study, we were limited to the tracts generated with the aforementioned limitations. We did not identify microcystic change that precluded targeting even in the patients with the lowest DRS scores.

In sum, we found that there was preserved nuclear structure of the NBM and identifiable efferent pathways in our PD patients. Therefore we believe that DTI can aid in targeting the nucleus and that targeting in PDD patients will not likely be affected by microcystic change seen in AD patients. We identified the anterolateral aspect of the NBM as having the densest connections to both the recipients of the medial pathway and the amygdala and temporal lobe. However, the parieto-occipital projections were found more postero-laterally. We would expect that if the stimulation induced effects are confined to the fibers stimulated that different cortical abilities would be affected and that improvements may be confined to these areas, e.g., constructional apraxia with parietal stimulation and/or language with posterior temporal fiber stimulation.

Both (Freund et al., 2009) and (Gratwicke et al., 2013) targeted Ch4i in PDD and saw the biggest effects on constructional apraxia and hallucinations both thought to be the result of loss of NBM-parietal connectivity. Hepp et al. (2017) performed DTI imaging in PD patients looking at FA and mean diffusivity values of the NBM outflow tracts. They found significant changes in mean diffusivity in the parieto-occipital projections of patients with hallucinations (Hepp et al., 2017). This would suggest that the targeted region in these 2 studies affected the parietal outflow tracts. It remains to be seen whether other cortical functions can be affected by NBM DBS. Undoubtedly, the inclusion of DTI in future studies will shed additional light on the topic.

## Qeeg as a Biomarker

Diffusion tensor imaging targeting guidance for placement of the DBS will provide improved anatomic targeting of the NBM, however, physiologic confirmation is still needed both in and out of the operating room. In contrast to motor targets where there is an immediate and visible change in the motor exam, programming of cognitive targets have been based entirely on theoretical concepts without immediate feedback. We propose that NBM induced changes in EEG activity can be utilized as a physiologic biomarker to guide targeting, programming, and serve as a secondary outcome measure.

Stimulation of NBM causes EEG desynchronization in the neocortex (Metherate et al., 1992; McLin et al., 2002b; Montero-Pastor et al., 2004), shifting the EEG power spectra from lower frequency, higher voltage theta and alpha waves to higher frequency gamma waves, indicative of a more active cognitive state. (Celesia and Jasper, 1966; Détári et al., 1997, 1999; Duque et al., 2000), and correlates with associative learning in the involved cortex.

This change in EEG activity has been utilized to identify the connected cortex during NBM stimulation in animal models. Weinberger and colleagues performed extensive studies on the effect of NBM stimulation on auditory learning, taking advantage of the tonotopic organization of the auditory cortex. They placed an EEG electrode over the tonotopic auditory cortex. After a 3 day recovery period they lowered an electrode toward the NBM coordinates for the auditory subnucleus, while stimulating, until EEG activation of the auditory cortex was identified. They found a significant decrease in the lower frequency bands of delta, theta, alpha, and beta1 with an increase in beta2 and gamma bands in the local EEG. This response to NBM stimulation has been previously reported in multiple other sources (Belardetti et al., 1977; Metherate et al., 1992; Cape and Jones, 1998).

Quantitative electroencephalography involves the mathematical processing of EEG data to allow derivative factors to be extracted from the EEG data. This provides objective data, in contrast to the visual qualitative interpretation of the standard EEG. Please see the excellent review article by Cozac et al. (2016). QEEG can be performed on standard EEG arrays of 16–21 EEG electrodes or dense array EEG, which utilizes 256 EEG electrodes. The electrodes can be mapped onto the patient’s brain MRI using optical registration methods to provide more accurate localization of the activity. This objective data can be trolled with machine learning algorithms to detect patterns of activation associated with circuit and perhaps sub-circuit activation.

This concept is not new, as QEEG has been previously utilized to identify responses to DBS. This has been particularly useful in identifying a response to stimulation where there is no immediate measurable motor effect such as in fornix or SCC stimulation but can also be correlated with motor effects in more traditional basal ganglia or thalamic stimulation for movement disorders. Laxton et al. (2010) utilized sLORETA to identify and map the brain areas that are affected by electrical stimulation of the fornix in AD patients. An evoked response was elicited to 3 Hz stimulation with a 450 μs pulse width. The evoked responses of 500 consecutive stimuli were averaged and compared with baseline EEG activity and were presented as images of statistically standardized current density distributions on a cortical grid of 6,239 voxels. They identified the resultant activation in the ipsilateral hippocampal formation and the medial temporal lobe. With longer latencies the activation shifts to the cingulate gyrus, and the precuneus. This data was consistent with the PET data in the same patients showing activation of the temporal lobe, posterior cingulate and medial parietal lobe, which are part of the default mode network. The response was dose-related correlating with the amplitude of stimulation (range 1–10 V). Broadway et al. (2012) employed QEEG to identify biomarkers for response of patients to subgenual cortex stimulation for the treatment of intractable depression. Differences in frontal theta current source density in the region of the ACC and PFC is associated with depression as well as response to treatment. This same effect is seen in the SCC on PET scanning as a hypermetabolic area and its return to normal with resolution of the depression. An additional method of analysis is the change in theta cordance. “Cordance is a linear combination of normalized absolute power and relative power in a specific frequency band at a specific recording site” (Broadway et al., 2012). These investigators obtained EEGs at baseline and 6 and 24 months after DBS implantation in the SCC in 12 patients with intractable depression. They found a quantitative correlation of the FTC with the magnitude of depression reduction. Lower FTC at baseline (and higher FTC after 4 weeks) predicted lower depression severity scores after 24 weeks. This was specific to the frontal electrodes and was not a generalized phenomenon.

Thus there is evidence that QEEG can be informative of the long term consequences of stimulation. It remains to be explored whether the QEEG can be used in a more immediate fashion to analyze the utility of different contacts placed in a target region. The utility and fidelity of this approach has not been tested in standard targets, however, there is preliminary evidence of the validity of this approach. Specifically, prior investigators have identified STN-DBS induced increased alpha activity in the temporo-parieto-occipital regions and mu-rhythm in the fronto-central area and ventral intermediate (VIM)-DBS induced desynchronization of alpha and theta activity in M1 and S1 respectively (Air et al., 2012; Jech et al., 2016). This can also be seen in the event related potentials as in Walker et al. (2012).

### Preliminary Original Data

**FIGURE 4:**
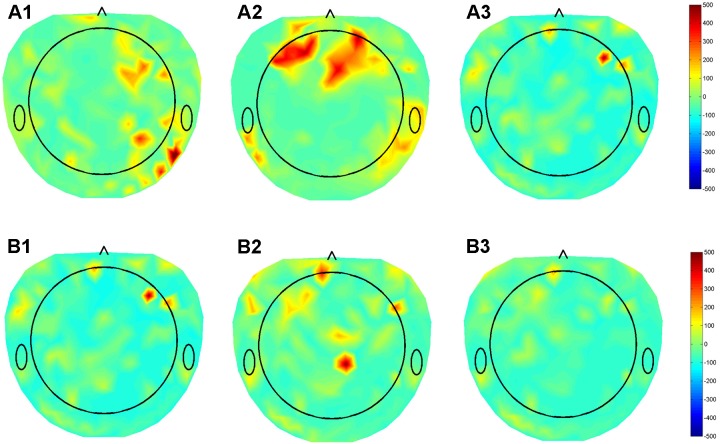
Topographical maps of QEEG from three patients with DBS leads in different nuclei (GPI, STN, VIM), using the Electrical Geodesics Inc. data acquisition system. **(A)** Subtraction plot of beta band (12–30 Hz) activity in the “On” minus “Off” stimulation conditions using optimal DBS lead configurations and parameters for in **(A1)** GPI **(A2)** STN **(A3)** VIM during the awake resting condition. **(B)** Topoplots for EEG beta band for single patient using the **(B1)** Optimal (1,2), **(B2)** contact 0, and **(B3)** contact 3 of DBS electrode demonstrating differential modulation of different sub-regions in the cortex.

We previously collected dense array EEG data from three patients with DBS leads in different nuclei (GPI, STN, VIM), using the Electrical Geodesics Inc. data acquisition system. The beta band (12–30 Hz) activity in the cortex was assessed in the on stimulation condition using the patient’s optimal settings and when the stimulator was turned off. The raw EEG data was preprocessed and corrected for non-physiological and movement artifacts. Additional spatial and temporal filtering enhanced local EEG features. Finally, the beta band power of the data segments collected during resting condition was calculated and the modulation in the beta band during DBS ON-OFF state was estimated for each of the 256 dense array EEG electrodes were calculated and a beta modulation topographical subtraction map representation were generated. This demonstrated very different patterns of activation for GPI, VIM, and STN providing preliminary evidence that this response may be used to identify the stimulated nucleus. We also stimulated the distal and proximal contacts of a single lead (**Figure 4**). Despite the relative proximity of these contacts, their differential activation resulted in clearly different cortical beta activity, suggesting that the EEG may be useful in differentiating the utility of each contact within a lead. Although much work remains to be done to prove the fidelity of these responses, we hypothesize that QEEG can be used as an objective tool to quantify real-time responses to DBS with high sensitivity to target location, and further correlation with the corresponding changes in phenotypic response (symptoms/side effects), and that this will be even more useful in cognitive nuclei such as NBM.

Thus, QEEG identification of the cortical area activated by NBM stimulation is feasible and would be expected to provide guidance for targeting and programming, as well as provide a secondary objective outcome measure. A comprehensive study is underway assessing alpha, theta, delta, and lower gamma bands, as well as smaller frequency bins (0.1–2 Hz) with the intent to identify differentially modulated territories by specific stimulation targets.

## Optimization of DBS Parameters

Nucleus basalis of Meynert stimulation primarily affects the neocortical environment by modulating cortical CBF and the discharge properties of the target nuclei through the release of ACh and NGF to alter the neocortical excitability.

## NBM Stimulation Induced Variations in CBF

The cortical blood flow (CBF) varies significantly based on the level of local neural activity as well as NBM activation of the area (Biesold et al., 1989; Adachi et al., 1992; Vaucher et al., 1997; Lacombe et al., 2006; Piché et al., 2010). The cholinergic nerve terminals of the NBM are in direct contact with the cortical parenchymal capillaries and arterioles allowing NBM activity or stimulation to increase CBF though vasodilation of these vessels (Hotta et al., 2004). Additionally, cortical nitric oxide synthase neurons and cortical GABAergic interneurons are mediators of the NBM cholinergic increases in CBF (Sato et al., 2001; Kocharyan et al., 2008). This control of CBF via the NBM is independent of regional cortical activity, cerebral metabolites, and blood pressure (Kurosawa et al., 1989; Sato et al., 2001) indicating that stimulation of NBM can independently control blood flow.

The NBM induced increase in CBF is maintained during NBM stimulation and returns to baseline after the stimulation is stopped (Hotta et al., 2004). CBF increases in a dose dependent manner based on the intensity and frequency of stimulation. Unilateral stimulation results in widespread ipsilateral hemispheric increase in blood flow. Vaucher et al. (1994) demonstrated that the cortical distribution of the CBF response is not specific to the area of NBM stimulation and is instead stereotypic in its response to stimulation of multiple areas within the NBM. Thus, the CBF response cannot serve as a marker of stimulation of the specific sub-regions of the NBM.

## NBM Stimulation Induced Variations in Neocortical Excitability

The optimal stimulation paradigm for neocortical excitability should augment and maintain the level of ACh in the cortex. This in turn, would provide a faciliatory platform for the neuroplastic integration of new sensory inputs within memory engrams (Weinberger et al., 2006, 2013). Despite being outnumbered by its cholinergic counterpart, the non-cholinergic (GABAergic and glutamatergic) cortico-petal population of the NBM plays a pivotal role in cortical arousal, plasticity, and the sleep–wake cycle. The mechanism of precisely how these GABAergic and glutamatergic projections interact with cholinergic signals in the BF is still poorly understood. However, a subset of GABAergic BF projections (parvalbumin positive, PV+) end on cortical GABAergic interneurons, and are selectively involved in modulating gamma oscillations and thus controlling the synchronization of cortical PV interneuron activity. In addition, the cortical input to the basal forebrain comes from the medial and lateral prefrontal and ventral orbitofrontal cortices, which synapses exclusively on the GABAergic neurons (Sarter and Bruno, 2002). Selective lesioning of the GABAergic population creates deficits in set switching, such that the rats perseverate on signals that were previously rewarded but no longer are. Thus, the GABAergic population may have a specific role in executive functioning and may provide the ability to switch attention from one task to the other. Another subset of BF GABAergic projections is somatostatin-positive (SOM+) neurons, which are thought to promote sleep (Xu et al., 2015; Do et al., 2016). Glutaminergic BF neurons form reciprocal connections with other nuclei (Do et al., 2016), particularly with the hypothalamus and striatum, but their role in memory remains largely unexplored. A group of phasically bursting non-cholinergic non-PV-GABAergic BF neurons (probably glutaminergic) are reported to increase the cortical representation of detected stimuli (Lin et al., 2006; Avila and Lin, 2014). The cholinergic and non-cholinergic BF/NBM neurons work synergistically to control the dynamic cortical modulation, where each subset is responsible for different aspects of the behavior (Lin et al., 2015). For instance, the cholinergic projections respond to reinforcement signals, whereas the non-cholinergic inputs respond to cues for reinforcement. Electrical stimulation of the NBM has been repeatedly shown to improve learning and attention but the relative effect of GABAergic and cholinergic contributions to each experimental paradigm is not only undefined, but also is likely task specific, and may also be dependent on the stimulation parameters.

The traditional electrical brain stimulation techniques in human are limited to simple tonic patterns, while adjusting the frequency, pulse width, and voltage for individual patients to maximize the observed efficacy. This has been effective in clinically silencing overactive nuclei, but may not be as effective at activating connected cortex for specific functions. In contrast, burst stimulation of NBM has been widely reported to boost its downstream cholinergic effects. Bursty phasic discharge patterns are abundant in the nervous system and are linked to many important neuronal phenomena including memory processes. Naturalistic memory formation is associated with burst firing patterns in the NBM during learning (Sarter et al., 2014).

In fact, most of NBM stimulation in animals has been conducted with short burst stimulation or a periodic combination of bursts with pauses between bursts. **Table 2** summarizes the NBM stimulation modes and patterns tested in various learning paradigms in the animal literature. These studies have demonstrated multiple factors that favor burst stimulation over tonic. Burst stimulation is more powerful in eliciting both excitatory and inhibitory postsynaptic potentials (EPSP and IPSP). Burst patterns carry more information than tonic signaling and are key components of network synchrony (Cain and Snutch, 2013). The NBM neurons that discharge in phasic burst mode are more excitable and respond more quickly to cortical inputs than tonic NBM neurons (Unal et al., 2012). Clearly, these ‘burst’ firing patterns are critical components of NBM function.

We are therefore challenged to devise a DBS stimulation paradigm that mimics and optimizes the naturalistic effects of NBM activity. We would suggest that this can be best accomplished by utilizing phasic burst stimulation patterns. In this section, we will discuss various technical and theoretical considerations to systematically design optimal parameters for burst stimulation of NBM for the treatment of dementia and related disorders. Throughout the text we utilize text boxes found in **Supplementary Materials** to illustrate and elaborate on the underlying principles of neural network principles that form the basis of this approach.

Cortical and subcortical gamma activities are markers of local network activity including learning and memory processes (Buzsáki and Wang, 2012). **Supplementary Figure S1** describes the origin of local gamma oscillations and the resultant resonance in this local network.

Strong gamma oscillations originating in any local assembly indicate coordinated neuronal activity, which reflects synaptic integration and modulation of post-synaptic neurons (Buzsáki and Wang, 2012). Moreover, the time scale of gamma power lies within the critical time window of spike dependent neuroplasticity (Bi and Poo, 1998; Wespatat et al., 2004; Isaac et al., 2009; Weinberger et al., 2013).

Network activity is composed of cortical areas with gamma activity oscillating in the same frequency. However, gamma oscillations that are out of phase are not allowed to participate. Input (presynaptic) discharges that are out of the gamma phase window will not generate adequate postsynaptic alterations to modulate the local network (Buzsáki et al., 2004; Buzsáki, 2010). However, information can be transferred between these areas, if their oscillations become synchronized or in phase through a process called phase locking. Modification of the oscillatory pattern of a targeted area of cortex to match the network resonant frequency allows the strong coupling between them for effective information transmission (Buehlmann and Deco, 2010; Lowet et al., 2016). **Supplementary Figure S2** explains how the susceptibility of neurons to inputs at a certain phase of the neuronal discharge can result in alteration in the neuron’s firing pattern to fall into synchrony with the input of an oscillatory pattern.

Whereas, high frequency activity (like gamma oscillations) subserves local networks, longer wavelength activity links larger networks. Frequencies such as delta, theta, and slower waves create larger field connections between widespread cortical sites. CFC (Buzsáki and Wang, 2012) of gamma with these slower frequency bands is the mechanism of integration of these two signals into one oscillatory pattern. This CFC allows reliable transfer of information between various fast oscillating (gamma) local networks that together form a larger network operating at slower timescales for more complex processes. This in turn will propagate to other coupled areas in the network via inter-regional phase locking. The CFC results in a periodic pattern of bursts with pauses between bursts. This same pattern has been utilized in the animal studies denoted by gamma-delta stimulation outlined in **Table 2**. **Supplementary Figure S3** depicts the origin of fast oscillations in local network, slow oscillation in large-scale NBM network, and the result of cross frequency coupling between these oscillations.

This phase locking is controlled by the connectivity of the cortex with the NBM (Sanchez-Alavez et al., 2014). The result is that the NBM drives the EEG-LFP spectrums in different activated subcortical and cortical regions (Buzsaki et al., 1988; Metherate et al., 1992; Lee et al., 2005; Kalmbach et al., 2012; Nguyen and Lin, 2014). Correspondingly, lesions in NBM are reported to cause reduced phase locking in gamma, delta and beta bands in frontal cortex and HC (Sanchez-Alavez et al., 2014).

During learning induced neuroplastic reorganization, the various cortical sub-circuitries are tuned by the NBM to allow participation in the response. Thus, phase locking of cross frequency coupled oscillations provides the basis for selective routing of information to certain cortical sub-networks (e.g., auditory cortex for audio task learning or barrel cortex for somatosensory learning or both for combined tasks). The degree to which this synchrony is achieved affects the end result. The degree of phase locked theta modulation of frontal and temporal lobe has been reported to be proportional to memory task performance. As an example, gamma power in the frontal and temporal regions are phase-locked to theta oscillations during an auditory task (Voytek et al., 2010). Similarly during visual attention, V1 and V4 oscillate in synchrony in a high frequency gamma (50–80 Hz) band, while theta oscillatory power shows a drastic increase in both parietal and occipital cortex during learning trials (Raghavachari et al., 2001, 2006; Voytek et al., 2010) indicating its role in memory encoding.

Nucleus basalis of Meynert stimulation can restore the ability of damaged degenerated networks to phase lock and therefore communicate. **Supplementary Figure S4** describes how burst NBM stimulation pattern can selectively alter the cortical processing to facilitate learning. In the normal healthy brain, the connectivity, i.e., the κ, between NBM and the neocortical oscillatory systems is strong. Based on the Arnold tongue (Pikovsky et al., 2003) curve, synchronized information transfer can occur even if the oscillatory frequencies of the two systems are not precisely tuned together, i.e., if the κ for the system is high. Thus, for regions with strong anatomic connectivity, a relatively broad range of oscillatory frequencies will still allow information transfer. However, precise tuning of the oscillatory frequency is required for minimally connected regions. In dementia, where the number of cells in the NBM has been drastically reduced, the κ between the NBM and neocortical targets is also reduced. Thus, the weak phasic oscillatory strength of the remaining NBM population may be not strong enough to modulate neocortical neurons to fire in synchrony with the source nuclei thereby allowing a phase precession. The information flow and the phase synchronization between the two systems can then further deteriorate. A strong external stimulation pattern within a precise frequency range may revive the synchronization within the networks through perfect phase locking. Thus NBM stimulation may not only serve to facilitate communication across intact networks, it may have the capacity to revitalize networks that have fallen out of phase and therefore out of use.

In summary, we can hypothesize that we can enhance the cortical response by optimizing the oscillatory frequency of a burst stimulation pattern. We propose that modulating the baseline gamma burst stimulation frequency, specifically with a slower rhythm such as theta or delta will pose more effective coupling between NBM and different cortical regions involved in many learning processes. The selectivity is created by the specific pathway that the stimulation is applied to, as well as the oscillatory pattern chosen.

We anticipate that this will allow us to strengthen the flow of information in a network weakened by disease.

## Regenerative Aspects of NBM DBS

NBM DBS is unique amongst DBS applications in that it targets the degenerating nucleus itself for the placement of the DBS electrodes. This is in contrast to all other DBS targets, which involve targeting downstream affected circuits. This approach provides a unique opportunity to assess the ability of electrical stimulation to protect or repair ongoing damage to the brain. In addition to the NBM stimulation induced cortical plasticity already discussed, we hypothesize that NBM DBS has the potential to also induce restorative neuroplastic changes via stimulation-induced neurogenesis and through NGF release with increased neuronal survival.

Electrical stimulation has been shown in multiple paradigms to increase neurogenesis. This has been primarily evaluated in the hippocampus with more limited evaluation of the SVZ and RMS. Stimulation of the anterior thalamus (Hamani et al., 2011), nucleus accumbens (Schmuckermair et al., 2013), ventromedial PFC (Bambico et al., 2015), entorhinal cortex (Stone et al., 2011), and medial septum (Jeong et al., 2014) have all been demonstrated to increase neurogenesis. Importantly neurogenesis has also been demonstrated in humans. Vedam-Mai et al. (2014) stained postmortem brains of Parkinson’s patients and found a 2–6 fold increase in neurogenesis in the SVZ of patients with DBS as compared to the PD patients without (Vedam-Mai et al., 2014). It is well recognized that neural progenitor cells will also migrate from the SVZ to a lesion (Saha et al., 2013; Capilla-Gonzalez et al., 2015) where they typically differentiate to glia and very few neurons. Endogenous electrical current exists within the brain and plays a role in directing neuroblast migration from the SVZ to the olfactory bulb along the RMS. Cao et al. (2013) demonstrated changes in the migratory direction of neuroblasts in culture and in brain slices (toward the cathode) with manipulation of an electrical field. The endogenous current measures 3–5 mV and they evaluated currents up to 250 mV, which is 1/100th of the voltage typically generated by a DBS. The electrical field can also favor neuronal differentiation of the neuroblast (Kobelt et al., 2014). Thus there is ample evidence that electrical fields play a role in neurogenesis and that neuroblast migration is directed toward the cathode of an induced electrical field. This is particularly relevant when the degenerating nucleus serves as the location of the implanted cathode and is in close proximity to the RMS. In this setting, it is also relevant to determine the ideal stimulation parameters to create neurogenesis and whether these correlate with effective stimulation for inducing behavioral improvement.

Nucleus basalis of Meynert activity normally affects the neocortical environment through the release of Ach, altering glial function, regulating cerebral blood flow, and inducing the secretion of NGF (Hotta et al., 2007). Glial cells such as astrocytes and microglia have been shown to respond NBM stimulation and play a major role in inducing synaptic plasticity. In response to stimulation, astrocytes produce higher levels of intracellular calcium and microglia release a variety of neuro-active compounds such as glutamate and D-serine. The combined effect facilitates *N*-methyl-D-aspartate receptor (NMDAR) dependent synaptic plasticity (Achour and Pascual, 2010; Takata et al., 2011). This plasticity did not occur in a knock out mouse (IP3 R2-KO) with absent astrocytic Ca surges. This suggests that glial cells in the neocortex play an important role in allowing synaptic plasticity (Takata et al., 2011).

In addition to influencing cortical neuronal and glial signaling, ACh stimulates the proliferation of NSCs and progenitor cells; thereby, playing an important role in neurogenesis (Ma et al., 2000; Gonzalez-Castaneda et al., 2011). While NBM ACh activity induces release of cortical NGF, NBM neurons, in turn, are dependent on cortical NGF for their survival (Whittemore and Seiger, 1987; Silver et al., 2001). Thus, the NBM and neocortex reciprocally support the functionality and survival of each other. To date, the ability of NBM stimulation-induced NGF release to reduce cell death in the face of a toxin has not been studied. We hypothesize that NBM stimulation and the resultant NGF release will correlate with sustained neuronal survival in NBM. Importantly, this effect will require longer timelines to evaluate in human trials and outcome assessments will need to extend out by several years to look for this effect. In the meanwhile, animal studies are ongoing in our laboratory.

## Role of Continuous Versus Activity Dependent NBM Stimulation

In addition to its role in cortical activation, the NBM also plays a role in the transition between sleep states (Lee et al., 2004). Cholinergic interneurons increase firing rates during wakefulness as well as REM (Irmak and Lecea, 2014). The activated NBM interneurons in turn, activate neighboring non-cholinergic cortically projecting GABA/parvalbumin (PV) neurons which promote arousal (Zant et al., 2016). Irmak and Lecea showed in a rat model that optogenetic stimulation of NBM cholinergic neurons is capable of transitioning an animal from NREM to a state of wakefulness and arousal within 20 s. In contrast, (Irmak and Lecea, 2014) stimulation during REM sleep was observed to prolong the REM state (Han et al., 2014). These experiments were conducted using short duration (5–20 s) optogenetic stimulation and so the effect of continuous stimulation throughout the sleep wake cycle remains to be studied.

A normal sleep wake cycle is important not only for the general health of an organism but also for its direct effects on the physiologic processes involved in learning and memory. Consolidation of verbal, spatial, and visual data, as well as skill acquisition and motor memory occurs during sleep (Fenn et al., 2003), and the experience dependent brain reactivation of memory traces during sleep have been shown in rodents, birds, humans, and nonhuman primates. Although the role of REM sleep in the consolidation of memory is controversial, the role of non-REM sleep is well established and thought to be critical (Buzsaki et al., 1988; Lee et al., 2004). After memory is successfully encoded, the supporting neuronal networks have been shown to repeat the neuronal activation to allow synaptic plasticity to consolidate the memory. These neural reactivations are seen during SWS, but very rarely during rapid eye-movement (REM) sleep (Dudai et al., 2015). This post-acquisitional reactivation has been correlated with successful memory acquisition in rats and humans (Ribeiro et al., 2004). Therefore, disruption in sleep-related memory consolidation could have deleterious effects on recall of learned information. It remains to be determined if continuous stimulation of the NBM during sleep may produce such deleterious effects.

The NBM also plays a role in resetting the circadian cycle through its connection with the SCN (Yamakawa et al., 2016). Most of the cholinergic inputs to the SCN are from the NBM and surrounding substantia innominata (Bina et al., 1993) and to a lesser extent from brainstem regions (Kiss and Halász, 1996; Castillo-Ruiz and Nunez, 2007). The effect of 24-h continuous NBM stimulation on the SCN and the circadian rhythm is unknown.

In summary, endogenous NBM activity plays a critical role in arousal, attention, and sleep cycle transitions. We, therefore, question the wisdom of 24-h continuous stimulation of the NBM and suggest that evaluation of these parameters is necessary.

## Alternative Potential DBS Targets for Treatment of Dementia

NBM (CH4), substantia innominata (SI), medial septum (MS, CH1), and horizontal limb of diagonal broca (CH3) have all been investigated for treatment of dementia (Linster and Hasselmo, 2001). This group of nuclei form a continuum of cholinergic neurons in the basal forebrain.(see **Table 1** for projections of these nuclei). SI is often used as a less specific term encompassing the NBM and surrounding nuclei that provides the major source of cholinergic projections to the cortex (Lamour et al., 1989); is associated with learning and attention (Fibiger and Phillips, 1986); and causes stimulation induced cortical vasodilation (Vaucher et al., 1994) and memory improvement. Similarly, MS-DBS has been reported to improve memory impairment caused by cholinergic denervation of the hippocampus. Electrical stimulation of the horizontal limb of the diagonal band of Broca (HDB) which projects to the main olfactory bulb (MOB) modulates evoke responses in the piriform cortex (Linster et al., 1999) and has a role in olfactory cognition and memory (Linster and Hasselmo, 2001) The NBM is severely affected in AD and PDD but the hippocampal projecting medial septal nucleus and vertical limb of the diagonal band (CH2) are not (Mufson et al., 1989).

In rodents, the NBM cells are scattered within and around the globus palidus, substantia innominata, and adjacent nuclei (Mesulam et al., 1983; McLin et al., 2002a). In humans, the anatomy of the NBM is somewhat more discrete, but the boundaries remain indistinct. Thus, the clinical effects of stimulation of this area will be determined by the volume of tissue activated (VTA) and whether the structures surrounding the NBM are involved. Thus, the potential to influence memory and cognition is not limited to the NBM and stimulation in this area has the potential to effect more than one of these structures. We have chosen to focus on the NBM because of its diffuse enervation of most of the cortex, allowing it to affect the broad range of cognitive functions that are affected by dementia.

## Conclusion

The NBM is a powerful nucleus, participating in almost all cortical networks, facilitating the incorporation of outside information into cortical engrams and their retrieval. This nucleus is slowly destroyed in PDD and AD. Stimulation of the NBM has the potential to restore functionality of this network, but it will require new clinical techniques to effectively translate the success in animal work to the human condition. Specifically, the diffuse connectivity of the nucleus is both a blessing and a curse. We must be able to target and identify the networks we engage and outcome measures must be chosen accordingly. We propose that DTI and QEEG can facilitate these endeavors. Next, we must think differently when we are attempting to activate, rather than deactivate, a network component and devise stimulation parameters that make theoretical sense and have the backing of proven efficacy in the animal literature. We must look to the effect not only in the EEG and short-term outcome measures, but also look for effects on connectivity, neurogenesis, and NGF-induced cortical preservation. Lastly, we cannot ignore the role of the NBM in the stages of sleep and the role of the stages of sleep on memory.

## Ethics Statement

This study was carried out in accordance with the recommendations of Veterans Health Administration, Good Clinical Practice guidelines, Department of Health and Human Services, Office of Human Research Protections by the McGuire Institutional Review Board (IRB) McGuire VA Medical Center. The protocol was approved by the McGuire Institutional Review Board (IRB), Research and Development Committee, McGuire VA Medical Center. All subjects gave written informed consent in accordance with the Declaration of Helsinki.

## Author Contributions

DK conceived and wrote sections on “Optimization of DBS Parameters” and “Regenerative Aspects of NBM DBS” and biomarker section. He was also involved in generating preliminary data for QEEG study. VP conceived and wrote “Targeting” section. He was also involved in generating preliminary data for the DTI section. JT contributed to the writing of the biomarker section. MM, KA, EH, and CW were involved in QEEG data analysis and topoplot generation. Additionally, EH provided technical assistance and manuscript editing. KH conceived and edited the entire manuscript and lead the study for the generation of preliminary data. All the authors were involved in editing the manuscript and approved the final version.

## Conflict of Interest Statement

The authors declare that the research was conducted in the absence of any commercial or financial relationships that could be construed as a potential conflict of interest.

## Supplementary Material

FIGURE S1 Simple model of origin of oscillations in neural system. The simplified neuronal model describes the effects of external perturbation and feedback loop from an interneuron. A self-pacing neuron-1 **(A1)** with no external inputs, spontaneously fires at a certain rate and phase **(A2)** dictated by its internal physiological mechanism. A single external pulse **(B1)** will change the phase of the upcoming spike **(B2)**. **(C1,2)** A simple neuron loop model describes the concept of resonance in neural network. The neuron-1 transmits an impulse (which can be self-initiated, paced or in response to other synaptic inputs) to neuron-2, which in turn feeds the signal back to neuron-1 **(C1)**. Based on their connectivity and phase relation, neuron-1 will now fire in an oscillatory pattern determined by the conductivity delay between neuron-1 and 2 **(C2)**. If the timing of the feedback impulse coincides with that of the impulse originating with neuron 1, the loop will resonate, i.e., the total summation at the axon hillock of neuron-1 will be stronger. The resonance frequency of this loop will depend upon the velocity of the nerve impulse passing through the fibers and synapses; and the length of the feedback loop. The effect of resonance will further be determined by intrinsic properties of neuron, type of neurotransmitter, their mechanism of action, and the negative or positive feedback.

FIGURE S2 Effect of stimulation at different phases on neuronal discharge pattern. Due to intrinsic and circuit resonance, the neurons will strongly respond to input pulses occurring at a certain phase of the neuronal discharge cycle. This box shows how the phase response curve (PRC) of a neuron is affected in the presence of an external random input. PRC characterizes the transient changes in the response of the target neuron. A neuron will fire based on the local network mechanism in the absence of any external stimulation **(A)**. If the perturbation is applied during a period of lower excitability, it will have lesser or negative impact on the target **(B)**. Whereas, if the perturbation occurs during a period of higher excitability, the target neuron is more sensitive to incoming spikes **(C,E)**. Depending upon the timing of the stimulation and the phase difference between the oscillatory target and the input, the resulting cycle will indicate either a phase advance **(C)** or phase delay **(E)**. If these perturbation are repetitive, the neuron will subsequently phase lock its firing with the incoming oscillatory stimulation **(D,E)**.

FIGURE S3 Origin of oscillations and resonance in NBM circuits. The schematic describes how multiple oscillatory frequencies are generated in NBM circuits **(A)**. The NBM forms a complex network within the memory circuit. The interneurons within the NBM form the local networks (green boxes in **A**). NBM also receives inputs from different limbic and paralimbic systems and relays the processed information to most of the neorcortex and amygdala (red boxes in **A**). At the local level, the GABAergic interneurons (out-2) provide negative feedback to the source NBM neuron (neuron-1). Similarly, within the cortico-NBM network, the cortical (e.g., entorihnal cortex etc.) and cortico-limbic systems echo their processed inputs (Out-4) back to the NBM forming a delayed negative feedback loop. Both of these local and inter-regional negative feedback loops cause an undershoot of NBM activity, which further reduces its excitatory input to cortex, resulting in a subsequent overshoot of NBM activity. This periodic undershoot and overshoot of activity gives rise to oscillations within the network. The conductance delay (D1, denoted by discrete-time operator, Z^−d1^) is shorter at the local level, resulting in a faster oscillatory frequency that is within in the gamma band (**B**, top panel). In contrast, the conductance delay (D2 denoted by discrete-time operator, Z^−d2^) is longer within the larger network level, generating slower oscillating frequencies, which range in the slow, delta, or theta bands (red trace in **B** bottom panel). The interaction of these complex interconnected networks and resultant adaptive closed loops gives rise to a dynamic pool of oscillatory activities within the network. The blue trace in bottom of **(B)** indicates the oscillatory burst pattern of a neuron in this network, where theta modulates the gamma oscillation of a local neuron. At the neuronal level, the cell fires in a burst mode, with an intra-burst frequency in the gamma range and an inter-burst pattern at the slower theta frequency (spike raster in bottom of **B**). Depending upon the current activity level, the delay factor of the feedback loop, type of neurotransmitter and the dispersion properties of the axon, each loop of the NBM-neocortical network will generate a propensity for or sensitivity to an oscillatory input of a certain frequency (Resonance). The local neuronal population will subsequently fire in phase with the network oscillations **(C)**, which can be observed in LFP’s in that region. Neurons in that network will show higher excitability for these oscillations and will efficiently phase lock their intrinsic firing in tune.

FIGURE S4 Oscillatory burst stimulation of NBM will selectively alter the neocortical response to facilitate learning. **(A)** Depicts a simplified computational flow chart of the mechanism of DBS in the NBM-cortical network, when NBM is stimulated with a pure burst oscillatory pattern. The internal network resonance mechanism acts as a band pass filter (blue panel), allowing only certain signal frequencies to pass, with the maximal response at the resonant input frequency of the network. If the burst oscillations are in the resonance range of the network, then based on the coupling strength, the neurons in the target nuclei will alter the timing of their spikes to oscillate in coherence with the input coupling frequency. This process is called phase locking and is dependent on the connectivity of the cortex with the NBM (Sanchez-Alavez et al., 2014). The mechanism of signal integration at the synapse, act as a phase detector (pink panel), which compares the phase of neuronal discharge (Oscil-1) with the phase of stimulation input. The difference in the phase drives the internal spiking to match the phase of the stimulation oscillations. Using this phase locking mechanism, strong NBM oscillatory stimulation **(C)** can drive the neurons of multiple local neocortical targets to spike in sync with it **(D)**. Modification of the slower oscillatory component (slow, delta or theta band) of the burst stimulation pattern to match the resonant frequency will allow strong coupling between multiple targets (Oscil-1 and Oscil-2)for effective information transmission via inter-regional coupling (Buehlmann and Deco, 2010; Lowet et al., 2016). Finally, the coupled NBM-cortical systems will have enhanced communication such that the intra-burst perturbation from NBM will be effectively reflected in the neocortical targets as well. Thus, the intra-burst gamma component of the stimulation pattern in these selected regions will then enhance ACh levels, facilitating neuronal plasticity for the incoming sensory signals (green panel in **A**).

## Abbreviations

AC: anterior commissure
ACC: anterior cingulate
ACh: acetylcholine
AD: Alzheimer’s disease
ADAS-cog: Alzheimer’s Disease Assessment Scale-cognitive subscale
ADC: apparent diffusion coefficient
ADL: activities of daily living
CFC: cross-frequency coupling
DBS: deep brain stimulation
DRS: dementia rating scale
DTI: diffusion tensor imaging
EPSP: excitatory postsynaptic potentials
FA: fractional anisotropy
FAST: Florida Apraxia Screening test
FTC: frontal theta cordance
GABA: gamma-aminobutyric acid
GPe: globus pallidus externa
GPi: globus pallidus interna
IPSP: inhibitory postsynaptic potentials
κ: coupling strength
LFP: local field potential
M1: primary motor cortex
MMSE: Mini- Mental State Examination
MRI: magnetic resonance imaging
MSE: Mental State Examination
NBM: nucleus basalis of Meynert
NGF: nerve growth factor
NPI: Neuropsychiatric Inventory
NREM: non-rapid eye movement sleep
NSCs: neural stem cells
PD: Parkinson’s disease
PDD: Parkinson’s disease dementia
PET: positron emission tomography
PFC: prefrontal cortex
PV: parvalbumin
QEEG: quantitative electroencephalography
REM: rapid eye movement sleep
RMS: rostral migratory stream
S1: primary somatosensory cortex
SCC: subgenual cingulate cortex
SCN: suprachiasmatic nucleus
sLORETA: standardized low-resolution electromagnetic tomography
STN: subthalamic nucleus
SVZ: subventricular zone
SWS: slow-wave sleep
VIM: ventral intermediate thalamus
VTA: volume of tissue activation.
