# Supplementary figures and images for: Nucleus Basalis of Meynert Stimulation for Dementia: Theoretical and Technical Considerations
Source: Front Neurosci. 2018 Sep 3;12:614. doi: 10.3389/fnins.2018.00614 (PMC6130053; doi:10.3389/fnins.2018.00614)

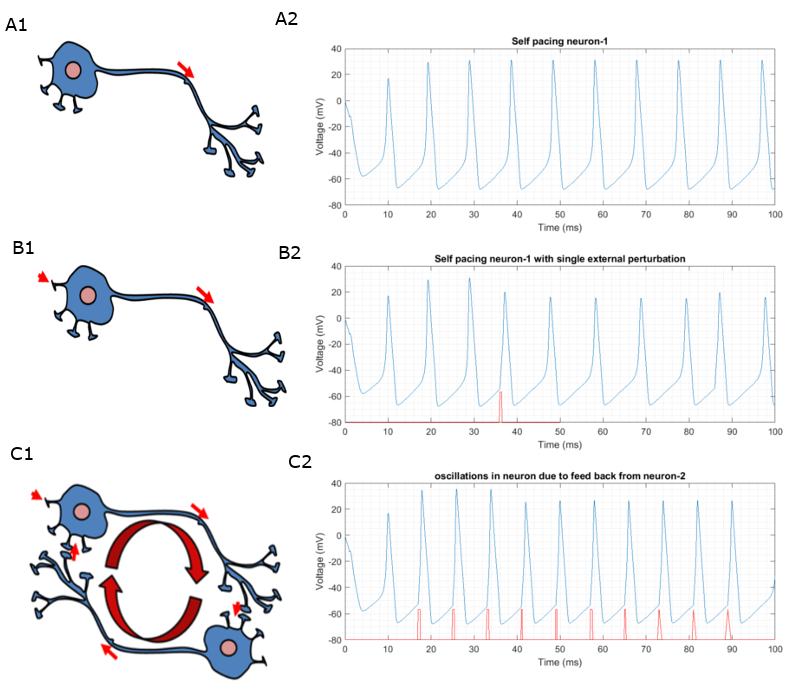

Supplement: FIGURE S1 — Simple model of origin of oscillations in neural system. The simplified neuronal model describes the effects of external perturbation and feedback loop from an interneuron. A self-pacing neuron-1 (A1) with no external inputs, spontaneously fires at a certain rate and phase (A2) dictated by its internal physiological mechanism. A single external pulse (B1) will change the phase of the upcoming spike (B2). (C1,2) A simple neuron loop model describes the concept of resonance in neural network. The neuron-1 transmits an impulse (which can be self-initiated, paced or in response to other synaptic inputs) to neuron-2, which in turn feeds the signal back to neuron-1 (C1). Based on their connectivity and phase relation, neuron-1 will now fire in an oscillatory pattern determined by the conductivity delay between neuron-1 and 2 (C2). If the timing of the feedback impulse coincides with that of the impulse originating with neuron 1, the loop will resonate, i.e., the total summation at the axon hillock of neuron-1 will be stronger. The resonance frequency of this loop will depend upon the velocity of the nerve impulse passing through the fibers and synapses; and the length of the feedback loop. The effect of resonance will further be determined by intrinsic properties of neuron, type of neurotransmitter, their mechanism of action, and the negative or positive feedback. [file Image_1.png]

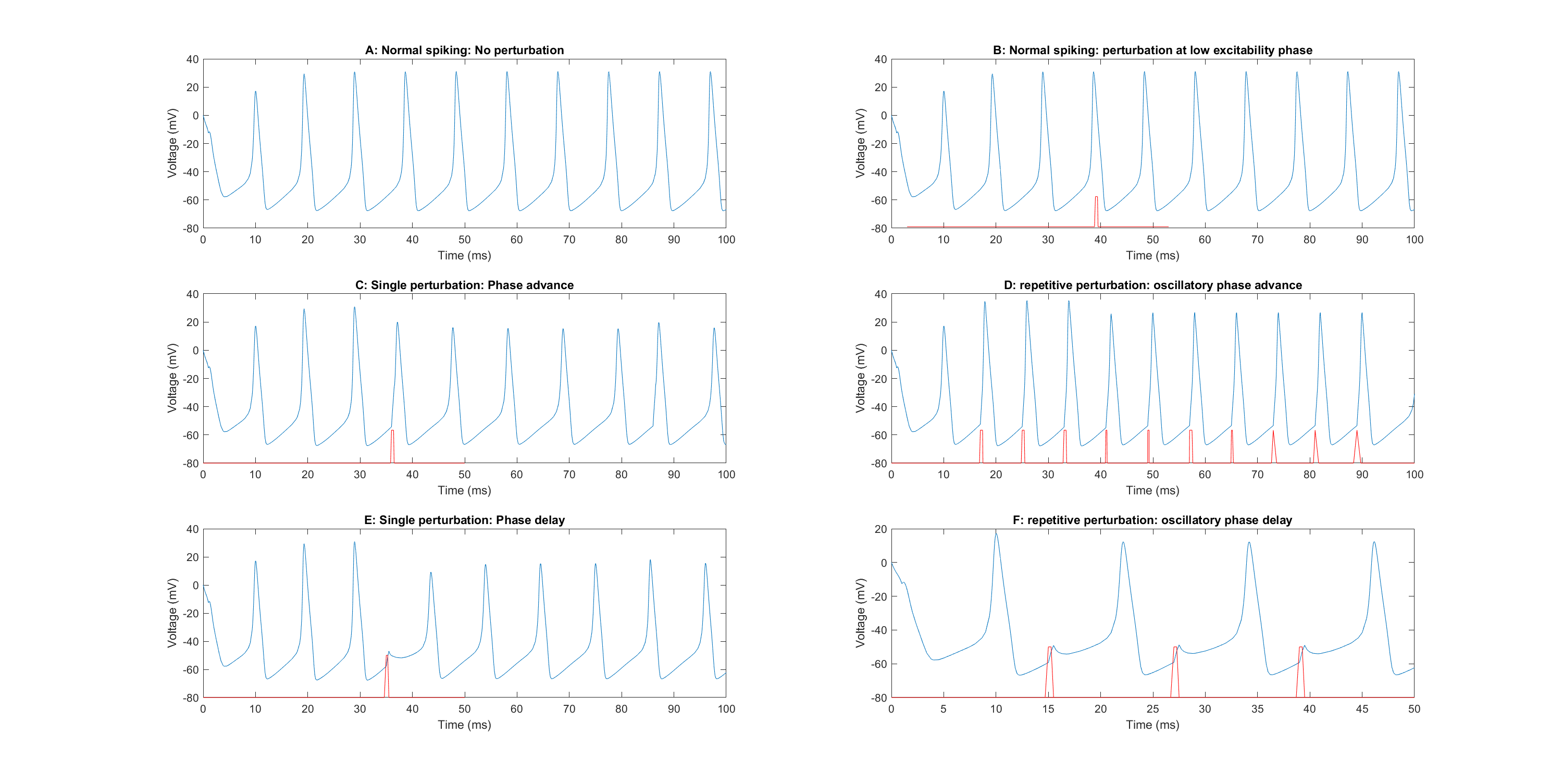

Supplement: FIGURE S2 — Effect of stimulation at different phases on neuronal discharge pattern. Due to intrinsic and circuit resonance, the neurons will strongly respond to input pulses occurring at a certain phase of the neuronal discharge cycle. This box shows how the phase response curve (PRC) of a neuron is affected in the presence of an external random input. PRC characterizes the transient changes in the response of the target neuron. A neuron will fire based on the local network mechanism in the absence of any external stimulation (A). If the perturbation is applied during a period of lower excitability, it will have lesser or negative impact on the target (B). Whereas, if the perturbation occurs during a period of higher excitability, the target neuron is more sensitive to incoming spikes (C,E). Depending upon the timing of the stimulation and the phase difference between the oscillatory target and the input, the resulting cycle will indicate either a phase advance (C) or phase delay (E). If these perturbation are repetitive, the neuron will subsequently phase lock its firing with the incoming oscillatory stimulation (D,E). [file Image_2.tif]

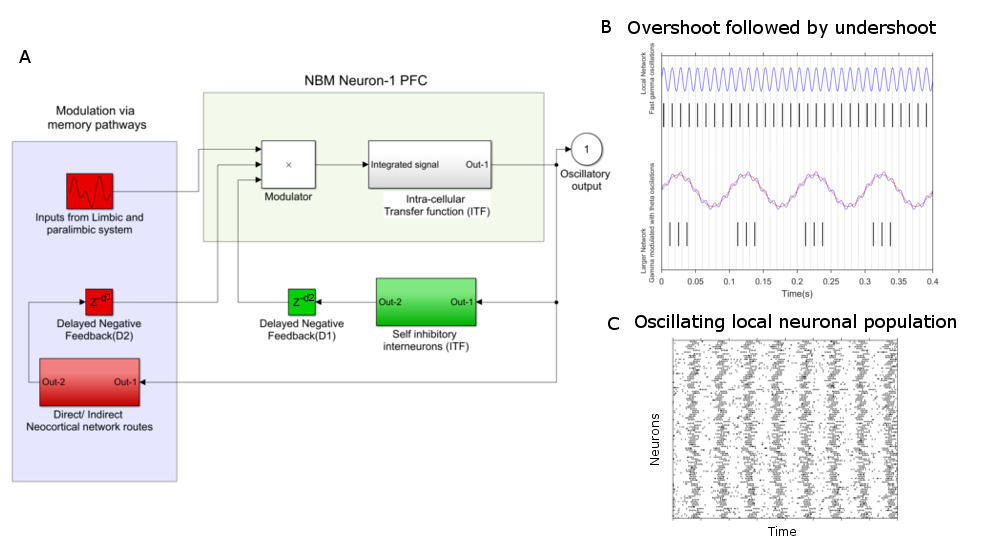

Supplement: FIGURE S3 — Origin of oscillations and resonance in NBM circuits. The schematic describes how multiple oscillatory frequencies are generated in NBM circuits (A). The NBM forms a complex network within the memory circuit. The interneurons within the NBM form the local networks (green boxes in A). NBM also receives inputs from different limbic and paralimbic systems and relays the processed information to most of the neorcortex and amygdala (red boxes in A). At the local level, the GABAergic interneurons (out-2) provide negative feedback to the source NBM neuron (neuron-1). Similarly, within the cortico-NBM network, the cortical (e.g., entorihnal cortex etc.) and cortico-limbic systems echo their processed inputs (Out-4) back to the NBM forming a delayed negative feedback loop. Both of these local and inter-regional negative feedback loops cause an undershoot of NBM activity, which further reduces its excitatory input to cortex, resulting in a subsequent overshoot of NBM activity. This periodic undershoot and overshoot of activity gives rise to oscillations within the network. The conductance delay (D1, denoted by discrete-time operator, Z−d1) is shorter at the local level, resulting in a faster oscillatory frequency that is within in the gamma band (B, top panel). In contrast, the conductance delay (D2 denoted by discrete-time operator, Z−d2) is longer within the larger network level, generating slower oscillating frequencies, which range in the slow, delta, or theta bands (red trace in B bottom panel). The interaction of these complex interconnected networks and resultant adaptive closed loops gives rise to a dynamic pool of oscillatory activities within the network. The blue trace in bottom of (B) indicates the oscillatory burst pattern of a neuron in this network, where theta modulates the gamma oscillation of a local neuron. At the neuronal level, the cell fires in a burst mode, with an intra-burst frequency in the gamma range and an inter-burst pattern at the slower theta f [file Image_3.tif]

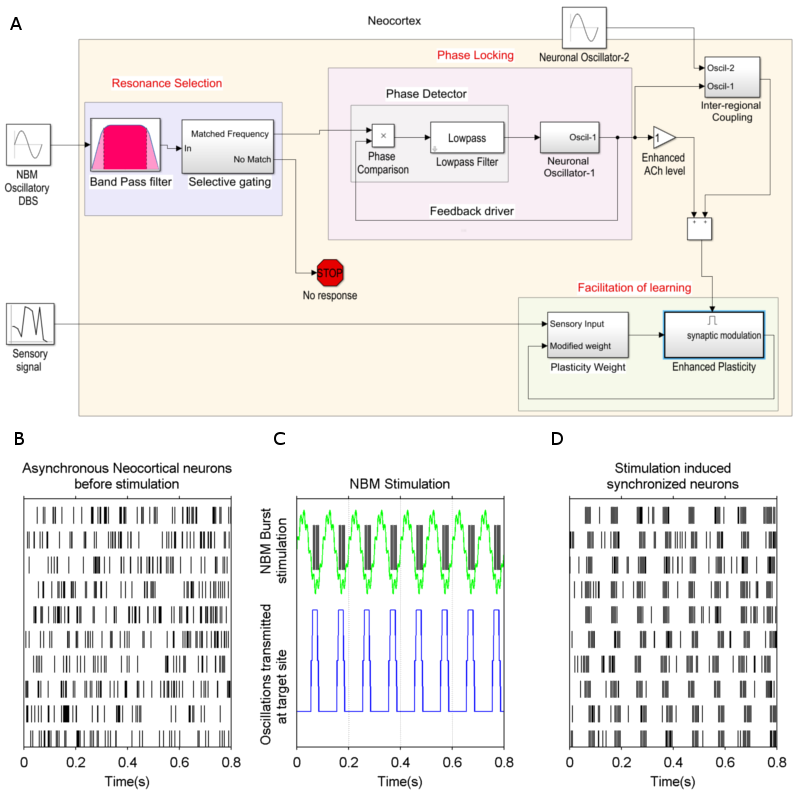

Supplement: FIGURE S4 — Oscillatory burst stimulation of NBM will selectively alter the neocortical response to facilitate learning. (A) Depicts a simplified computational flow chart of the mechanism of DBS in the NBM-cortical network, when NBM is stimulated with a pure burst oscillatory pattern. The internal network resonance mechanism acts as a band pass filter (blue panel), allowing only certain signal frequencies to pass, with the maximal response at the resonant input frequency of the network. If the burst oscillations are in the resonance range of the network, then based on the coupling strength, the neurons in the target nuclei will alter the timing of their spikes to oscillate in coherence with the input coupling frequency. This process is called phase locking and is dependent on the connectivity of the cortex with the NBM (Sanchez-Alavez et al., 2014). The mechanism of signal integration at the synapse, act as a phase detector (pink panel), which compares the phase of neuronal discharge (Oscil-1) with the phase of stimulation input. The difference in the phase drives the internal spiking to match the phase of the stimulation oscillations. Using this phase locking mechanism, strong NBM oscillatory stimulation (C) can drive the neurons of multiple local neocortical targets to spike in sync with it (D). Modification of the slower oscillatory component (slow, delta or theta band) of the burst stimulation pattern to match the resonant frequency will allow strong coupling between multiple targets (Oscil-1 and Oscil-2)for effective information transmission via inter-regional coupling (Buehlmann and Deco, 2010; Lowet et al., 2016). Finally, the coupled NBM-cortical systems will have enhanced communication such that the intra-burst perturbation from NBM will be effectively reflected in the neocortical targets as well. Thus, the intra-burst gamma component of the stimulation pattern in these selected regions will then enhance ACh levels, facilitating neuronal plasticity for the incoming sen [file Image_4.TIF]
